# Neuropathic Pain in Rats with a Partial Sciatic Nerve Ligation Is Alleviated by Intravenous Injection of Monoclonal Antibody to High Mobility Group Box-1

**DOI:** 10.1371/journal.pone.0073640

**Published:** 2013-08-21

**Authors:** Yoki Nakamura, Norimitsu Morioka, Hiromi Abe, Fang Fang Zhang, Kazue Hisaoka-Nakashima, Keyue Liu, Masahiro Nishibori, Yoshihiro Nakata

**Affiliations:** 1 Department of Pharmacology, Graduate School of Biomedical & Health Sciences, Hiroshima University, Hiroshima, Japan; 2 Department of Pharmacology, Graduate School of Medicine, Dentistry and Pharmacological Sciences, Okayama University, Okayama, Japan; Hokkaido University, Japan

## Abstract

High mobility group box-1 (HMGB1) is associated with the pathogenesis of inflammatory diseases. A previous study reported that intravenous injection of anti-HMGB1 monoclonal antibody significantly attenuated brain edema in a rat model of stroke, possibly by attenuating glial activation. Peripheral nerve injury leads to increased activity of glia in the spinal cord dorsal horn. Thus, it is possible that the anti-HMGB1 antibody could also be efficacious in attenuating peripheral nerve injury-induced pain. Following partial sciatic nerve ligation (PSNL), rats were treated with either anti-HMGB1 or control IgG. Intravenous treatment with anti-HMGB1 monoclonal antibody (2 mg/kg) significantly ameliorated PSNL-induced hind paw tactile hypersensitivity at 7, 14 and 21 days, but not 3 days, after ligation, whereas control IgG had no effect on tactile hypersensitivity. The expression of HMGB1 protein in the spinal dorsal horn was significantly increased 7, 14 and 21 days after PSNL; the efficacy of the anti-HMGB1 antibody is likely related to the presence of HMGB1 protein. Also, the injury-induced translocation of HMGB1 from the nucleus to the cytosol occurred mainly in dorsal horn neurons and not in astrocytes and microglia, indicating a neuronal source of HMGB1. Markers of astrocyte (glial fibrillary acidic protein (GFAP)), microglia (ionized calcium binding adaptor molecule 1 (Iba1)) and spinal neuron (cFos) activity were greatly increased in the ipsilateral dorsal horn side compared to the sham-operated side 21 days after PSNL. Anti-HMGB1 monoclonal antibody treatment significantly decreased the injury-induced expression of cFos and Iba1, but not GFAP. The results demonstrate that nerve injury evokes the synthesis and release of HMGB1 from spinal neurons, facilitating the activity of both microglia and neurons, which in turn leads to symptoms of neuropathic pain. Thus, the targeting of HMGB1 could be a useful therapeutic strategy in the treatment of chronic pain.

## Introduction

High mobility group box-1 (HMGB1) is considered to be a ubiquitous and abundant nonhistone DNA-binding protein, found in the nuclei of various cell types including neurons and glial cells [1]. While HMGB1 is a nuclear protein, interestingly, HMGB1 demonstrates cytokine-like effects in the extracellular space. A proinflammatory function of HMGB1 has been shown in several inflammatory disease states, including sepsis, acute lung injury, rheumatoid arthritis, amyotrophic lateral sclerosis and brain ischemia [2]–[8]. Previous studies reported that various inflammatory diseases, including brain infarction induced by the middle cerebral artery occlusion, brain edema induced by the traumatic brain injury and diet-induced atherosclerosis, were significantly ameliorated by treatment with an anti-HMGB1 monoclonal antibody that neutralizes HMGB1 peptides [7], [9]–[11]. Therefore, an anti-HMGB1 monoclonal antibody could be a potent therapeutic for inflammatory diseases [12]. Moreover, recent studies reported that HMGB1 in rodent spinal cord dorsal horn and dorsal root ganglion (DRG) plays a critical role in several animal models of chronic pain including diabetic, cancer and neuropathic pain [13]–[16]. To confirm a pro-nociceptive role of HMGB1, application of HMGB1 to the rat sciatic nerve evoked an enhanced sensitivity of the hind paw to both noxious and innocuous stimulation (hyperalgesia and allodynia, respectively) [15]. These data suggest that peripherally expressed HMGB1 can significantly modulate nociceptive processing.

There is accumulating evidence that spinal glial cells play a critical role in the formation of neuronal networks in the central nervous system [17]–[19]. Recent studies have clearly shown that spinal dorsal horn microglia and astrocyte are activated in the neuropathic pain state [20], [21]. Several neuropathic pain models have shown increased expression of microglia and astrocyte markers, including ionized calcium binding adaptor molecule 1 (Iba1) and glial fibrillary acidic protein (GFAP), respectively, in the dorsal horn [22], [23]. Activation of glial cells leads to the production and releases of a variety of inflammatory mediators, including cytokines, eicosanoids, neurotrophins and nitric oxide, which in turn induce nociceptive responses [18], [24]–[28]. While both microglia and astrocyte are activated following injury or in response to disease, it is possible that these cells have distinct roles in the pathology of neuropathic pain [17].

An animal model developed to study neuropathic pain is the partial sciatic nerve ligation (PSNL) model, which mimics some of the major features observed in clinical neuropathic pain [29]. Studies have reported an increased permeability of the blood spinal cord barrier (BSCB) to tracers such as Evans blue and sodium fluorescein, which was restricted to the lumbar spinal cord, which began 3 days after PSNL and lasted for at least 4 weeks following PSNL. Also, injury to a peripheral nerve and electrical stimulation of C-fibers each caused an increase in the permeability of the BSCB [30], [31]. Thus, in the PSNL model, large molecules, including antibodies, and immune cells can penetrate into or leak from the spinal cord, which suggests that a breakdown of BSCB is critical in the development of neuropathic pain. At the same time, the permeability of the BSCB following nerve injury could serve as a doorway into the spinal cord for large molecule-therapeutics.

It has been reported that a single injection with neutralizing anti-HMGB1 antibody into spinal cord attenuated diabetes-induced hypersensitivity in mice [14]. Thus, HMGB1 in spinal cord plays an important role in modulating pain sensation. In addition, a recent study showed that peripheral treatment with antibody attenuated subsequent mechanical allodynia in a rodent neuropathic pain model [15]. In that study, antibody was applied continuously to the injured nerve root for 7 days via an osmotic minipump. However, the effect of a systemic, acute treatment with anti-HMGB1 monoclonal antibody on neuropathic pain and the mechanism underlying the pro-nociceptive effect of HMGB1 in the spinal dorsal horn has yet to be determined. Systemic treatment with a neutralizing antibody could neutralize not only HMGB1 expressed in injured peripheral nerves and circulating HMGB1 but also that found in the spinal parenchyma. Hence, systemic treatment could also be beneficial for treating neuropathic pain.

The current study investigated the behavioral effect of intravenous (i.v.) injection of an anti-HMGB1 monoclonal antibody in rats with a PSNL. In addition, the current study elaborated the expression of HMGB1 in the spinal dorsal horn following PSNL and, furthermore, elucidated the mechanism of the antinociceptive effect of the antibody, by observing the activity of dorsal horn neurons and glial cells following antibody treatment.

## Materials and Methods

### Animals and animal care

Male Wistar rats, 5 to 8 weeks of age, were purchased from SHIMIZU Laboratory Supplies Co., Ltd. Rats were housed at a room temperature of 22±2°C with a 12 h light/dark cycle (lights on from 8:00 A.M. to 8:00 P.M.) and given access to food and water available ad libitum during the experimental period. Animal experiments were conducted in accordance with the “Guidelines for the Care and Use of Laboratory Animals” established by Hiroshima University, and all experimental procedures involving animals were approved by the Committee of Research Facilities for Laboratory Animal Science of Hiroshima University (Permit Number: A10-2-2).

### Partial sciatic nerve ligation model

The rats were anesthetized with sodium pentobarbital (50 mg/kg, i.p.). To induce a unilateral mononeuropathy, a tight ligation of approximately one-third to one-half of the diameter of the left sciatic nerve (ipsilateral) was performed with 6-0 silk suture as described previously [29]. In sham-operated rats, the nerve was exposed without ligation.

### Hind paw sensitivity to tactile stimulation

Three, 7, 14 and 21 days post-surgery, the plantar surface of the hind paws was probed with von Frey monofilaments (Natsume Seisakusho Co., Tokyo, Japan), starting with the filament that exerts 0.16 g of force. When the animal responded to the filament (which included lifting and licking of the hind paw), a lower force von Frey filament was used. If the animal did not respond, a higher force von Frey filament was applied. Each hind paw was tested three times. Either an anti-HMGB1 monoclonal antibody (IgG_2_a subclass, 2 mg/kg) or class-matched control monoclonal antibody (anti-*Keyhole Limpet hemocyanin*) was administered intravenously immediately following baseline withdrawal threshold determination.

### Western blot

Under ether anesthesia, rats were decapitated, the lumbar (L4–L6) segments of the spinal dorsal horn were removed and immediately frozen in liquid nitrogen and stored at –80°C until use. Spinal tissues were solubilized in radioimmunoprecipitation assay (RIPA) buffer with protease inhibitors (100 mM Tris–HCl, pH 7.4, 150 mM NaCl, 1 mM EDTA, 1% Triton X-100, 1% sodium deoxycholate, 0.1% sodium dodecyl sulfate (SDS), 20 µg/ml aprotinin, 20 µg/ml leupeptin, and 1 mM phenylmethylsulfonyl fluoride). The tissue lysates were then added to Laemli’s buffer and boiled for 5 min. Equal amounts of protein were separated by 12.5% SDS–polyacrylamide gel electrophoresis and blotted onto nitrocellulose membranes. The membranes were blocked in blocking buffer (20% skim milk in Tris buffered saline with Tween 20) for 2 h at room temperature, and subsequently incubated with a purified horseradish peroxidase (HRP)-conjugated monoclonal antibody against HMGB1 (1∶500), a monoclonal antibody against GFAP (1∶2,000), a polyclonal antibody against Iba1 (1∶1,000), a polyclonal antibody against cFos (1∶1,000) or a monoclonal antibody against β-actin (1∶20,000) overnight at 4°C. After washing, the membranes were incubated with a HRP-linked anti-mouse IgG antibody (1∶2,500) or a HRP-linked anti-rabbit secondary antibody (1∶2,500) for 1 h at room temperature. The membranes were then rinsed and incubated with Luminescence reagent (Thermo Fisher Scientific, Rockford, IL, USA). Finally, the membranes were exposed to X-ray film to detect the protein. For quantification of signals, the densities of specific bands were measured with Science Lab Image Gauge (Fuji Film, Tokyo, Japan). Specific bands for GFAP, Iba1, HMGB1, cFos and β -actin were detected at 51, 17, 30, 62 and 42 kDa, respectively.

### Immunohistochemistry

Under sodium pentobarbital anesthesia (50 mg/kg, i.p), rats were transcardially perfused with 50 ml of saline followed by 150 ml of freshly prepared 4% (w/v) paraformaldehyde in 0.1 M phosphate buffer (pH = 7.4). The spinal cord tissue was quickly removed and postfixed in the same fixative solution for three days at 4°C and then cryoprotected overnight in 30% (w/v) sucrose in 0.1 M phosphate buffer at 4°C. The tissue was embedded in Tissue-Tek OCT compound 4583 (Sakura Finetech, Tokyo, Japan) and frozen in liquid nitrogen, cut serially (30 µm thickness) in a cryostat, and collected onto glass slides (MAS-GP type A; Matsunami Glass, Osaka, Japan). After slides were dried at room temperature, tissue sections were processed for immunohistochemistry for cFos or double-labeling immunohistochemistry for HMGB1 and NeuN, HMGB1 and GFAP, HMGB1 and Iba1. The tissue sections were rinsed with phosphate-buffered saline, incubated in a blocking solution of 10% goat serum, 3% bovine serum albumin, 0.1% Triton X and 0.05% Tween-20 in phosphate-buffered saline for 2 h at room temperature, and then incubated with a mixture of mouse anti-GFAP antibody (1∶1,000), mouse anti-HMGB1 antibody (1∶250), rabbit anti-Iba1 antibody (1∶1,000), rabbit anti-NeuN antibody (1∶1,000), rabbit anti-GFAP antibody (1∶1,000) and rabbit anti-cFos antibody (1∶1,000) for 72 h at 4°C, followed by 4',6-diamidino-2-phenylindole (DAPI, 0.5 µg/mL) and corresponding secondary antibodies conjugated with Alexa Fluor® 488 (1∶200) and 555 (1∶250) for 2 h at 4°C in a dark chamber. The sections were then extensively washed in phosphate-buffered saline and then coverslipped. Sections were examined with a BZ-9000 Biorevo all-in-one fluorescence microscope (Keyence, Elmwood Park, NJ, USA). For quantification of signals, both immunoreactive intensity and area were measured with Image J software (NIH, Bethesda, USA).

Because of the technical difficulty in dividing tissue samples taken from experimental animals into cytosolic and nuclear fractions and at the same time determining the cell type in which translocation occurs HMGB1, we have devised a simple method of quantifying HMGB1 translocation as observed by immunocytochemistry. Cytoplasmic translocation of HMGB1 should result in an area of immunofluorescence that is greater than the area of DAPI, or non-translocated, nuclear HMGB1, immunofluorescence. Thus, as a measure of *in vivo* HMGB1 translocation, the ratio of the area of HMGB1 immunofluorescence to the area of DAPI immunofluorescence (as assessed using Image J) was calculated and this method was applied to glia as well as neurons. A value of 100 indicates nuclear HMGB1 (or a lack of cytoplasmic translocation) whereas values greater than 100 indicate cytoplasmic translocation of HMGB1.

## Materials

Anti-mouse IgG HRP-linked antibody (Cat. #7074S, Lot. #H0111) and anti-rabbit IgG HRP-linked antibody (Cat. #7074S, Lot. #C2812; Cell Signaling Technology, Beverly, MA, USA); Pentobarbital (Dainippon Sumitomo Pharma, Osaka, Japan); anti-rabbit GFAP antibody (Cat. #GTX72747, Lot. #14496; Gene Tex, Irvine, CA, USA); goat anti-rabbit IgG antibody (Alexa Fluor 555, Cat. #A-21428, Lot. #853493) and goat anti-mouse IgG antibody (Alexa Fluor 488, Cat. #A-11001, Lot. #877595; Invitrogen, Burlington, ON, Canada); anti-mouse GFAP antibody (Cat. #MAB360, Lot. #LV1718394) and anti-rabbit NeuN antibody (Cat. #MAB377, Lot. #1949597; Millipore Corporation, Bedford, MA, USA); bovine serum albumin (Nissui, Tokyo, Japan); DAPI (Santa Cruz Biotechnology, Santa Cruz, CA, USA); anti-mouse β-actin antibody (Cat. #A5441, Lot. #055K4854; Sigma-Aldrich Co., St. Louis, MO, USA) and anti-rabbit Iba1 antibody (Cat. #019-19741, Lot. #WEK6254; Wako Pure Chemical Industries, Osaka, Japan). All other reagents were of the highest purity available from commercial sources. The anti-HMGB1 rat monoclonal antibody was produced as described previously [7], [9].

### Statistical analysis

Values are expressed as mean ± SEM. Comparisons of expression of the spinal dorsal horn HMGB1 protein was performed using Student’s *t*-test. The data from the PSNL induced-mechanical hypersensitivity were analyzed using two-way analysis of variance (ANOVA) followed by the Tukey-Kramer test for *post hoc* comparisons, while data from the other experiments (comparisons between treatments) were analyzed using a one-way ANOVA followed by the Tukey-Kramer test for *post hoc* comparisons. A probability value (p) of less than 0.05 was considered to be statistically significant.

## Results

### PSNL evoked mechanical hypersensitivity and activation of spinal glial cells

**Figure 1:**
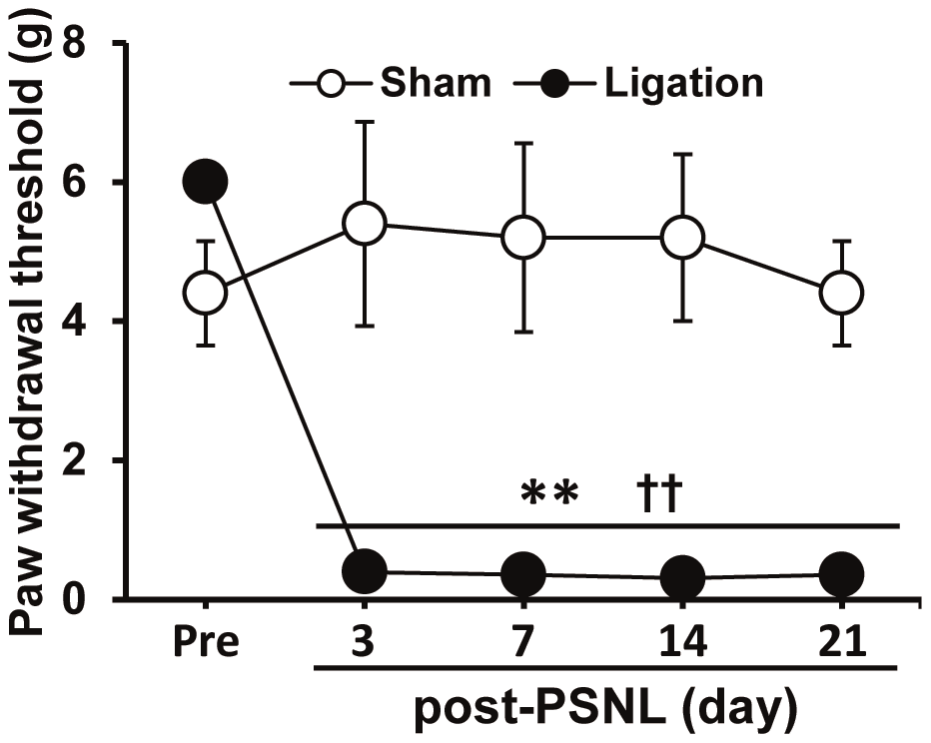
Partial Sciatic Nerve Ligation (PSNL) produces hind paw mechanical hypersensitivity in rats. Withdrawal thresholds of the ipsilateral hind paw following PSNL were assessed over time with von Frey filaments in sham and PSNL rats for the periods indicated. Data are expressed as mean ± SEM. n = 5/group. ** p<0.01 compared with pre-surgery (one-way ANOVA followed by Tukey-Kramer post hoc test). †† p<0.01 compared with sham group (Student’s t-test).

The development of long-lasting mechanical hypersensitivity in the left hind paw of PSNL rats was observed for 21 days after surgery (F_1, 40_ = 5.61, p<0.01, two-way ANOVA for repeated measures; Fig. 1). Rats with a PSNL had significantly lower withdrawal thresholds, compared with pre- surgery thresholds, of the ipsilateral paw at every time point after surgery (PSNL, pre: 6.00±0.00 g, 3 days: 0.39±0.07 g, 7 days: 0.35±0.05 g, 14 days: 0.30±0.06 g, 21 days: 0.35±0.05 g). By contrast, sham-operated rats did not display significant mechanical hypersensitivity (Sham, pre: 4.40±0.75 g, 3 days: 5.40±1.47 g, 7 days: 5.20±1.36 g, 14 days: 5.20±1.20 g, 21 days: 4.40±0.75 g,).

The rats displayed good overall health after either PSNL or sham surgery, with standard locomotor activity throughout the experimental period of evaluation.

### Effect of systemic treatment with anti-HMGB1 monoclonal antibody on PSNL-evoked mechanical hypersensitivity

**Figure 2:**
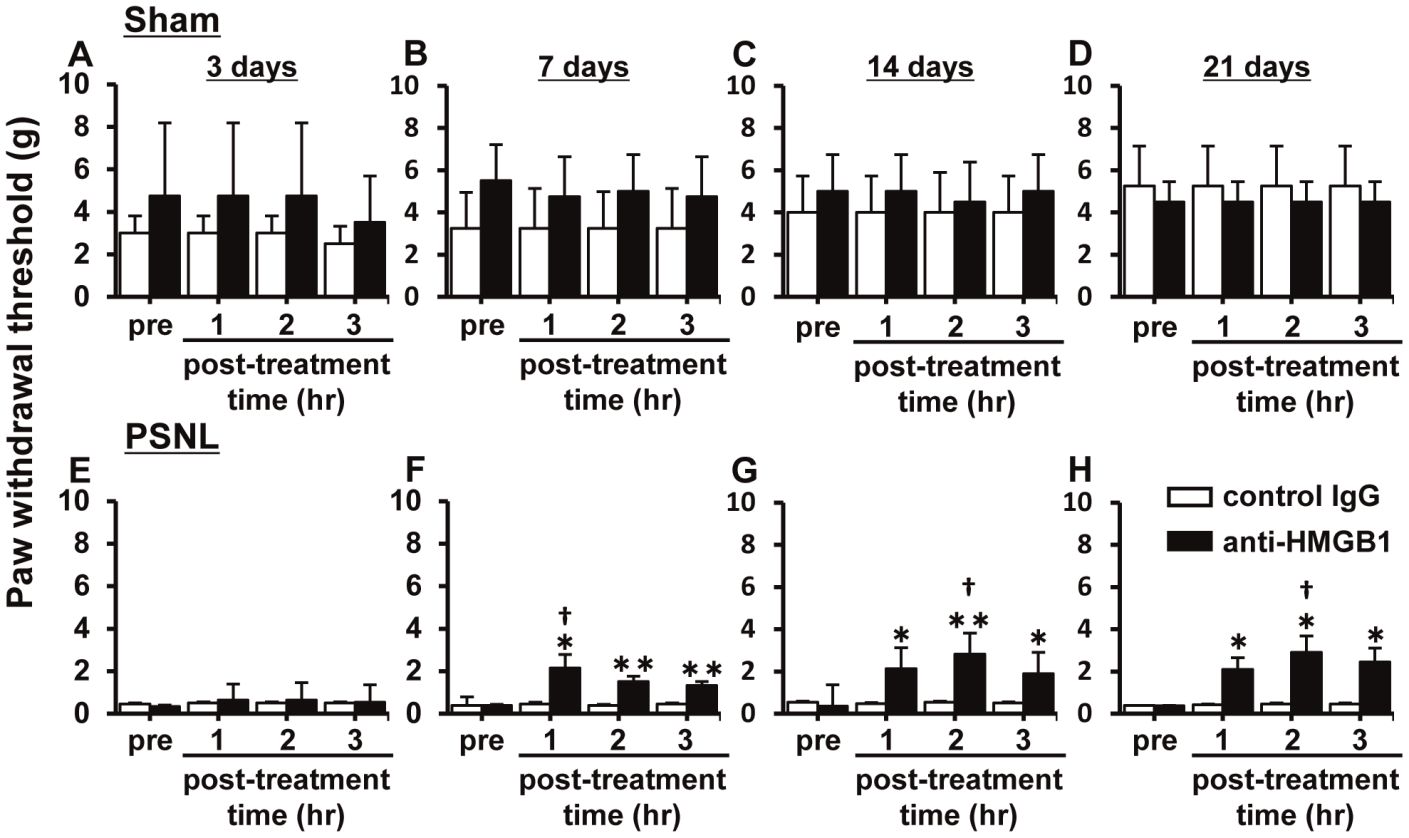
Antinociceptive effect of anti-HMGB1 monoclonal antibody on PSNL-induced mechanical hypersensitivity in rats. Effects of intravenous injection of either anti-HMGB1 monoclonal antibody (anti-HMGB1, 2 mg/kg) or control IgG (2 mg/kg) on nociceptive behaviors over time in rats with either sham or PSNL surgery (A, E: 3 days, B, F: 7 days, C, G: 14 days, D, H: 21 days). A single dose of anti-HMGB1 monoclonal antibody increased withdrawal thresholds in rats with a PSNL, but not sham, surgery. Data are expressed as mean ± SEM. n = 5/group. * p<0.05, ** p<0.01 compared with sham group (Student’s t-test). † p<0.05 compared with pre-operation (one-way ANOVA followed by Tukey-Kramer post hoc test).

As shown in Figure 2, systemic administration with an anti-HMGB1 monoclonal antibody (anti-HMGB1, 2 mg/kg, i.v.) significantly attenuated mechanical hypersensitivity induced by PSNL on days 7, 14, and 21, but not 3 days, post-surgery. The antinociceptive effect lasted for at least 3 hr after i.v. administration. By contrast, treatment with a control IgG antibody (2 mg/kg, i.v.) had no effect on withdrawal thresholds.

### HMGB1 up-regulation induced by PSNL surgery in spinal dorsal horn

**Figure 3:**
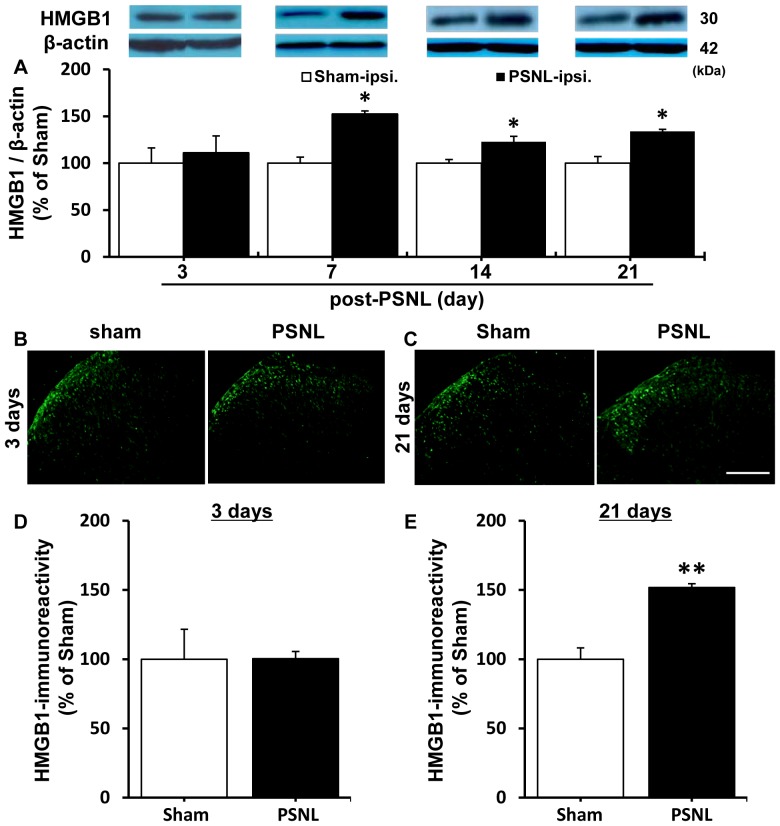
Expression level of HMGB1 was increased in rat lumbar spinal cord dorsal horn after PSNL. A. The expression of HMGB1 in the ipsilateral spinal dorsal horn of sham (post-Sham 3–21 days) and PSNL (post-PSNL 3–21 days) rats at the indicated periods were measured by Western blotting. The optic densities of HMGB1 were normalized to that of sham rats at the corresponding time point. n = 4/group. B, C. Immunolabeling of HMGB1 within spinal dorsal horn of sham and PSNL rats at 3 (B) and 21 days (C) following operation. Scale bar  =  200 µm. D, E. Quantitative analysis of HMGB1 expression at 3 (D) and 21 days (E) following operation. Data indicate the relative mean immunofluorescence intensity of spinal dorsal horn. Data are expressed as mean ± SEM. n = 3/group. *, ** p<0.05, 0.01 compared with sham group (Student’s t-test).

The expression levels of HMGB1 were determined on days 3, 7, 14 and 21 after PSNL by Western blotting. As shown in Figure 3A, levels of HMGB1 in ipsilateral dorsal horn were significantly elevated 7, 14 and 21 days, but not 3 days, after PSNL compared with levels of HMGB1 in dorsal horn from sham-operated rats. By contrast, PSNL surgery did not significantly affect expression levels of HMGB1 mRNA in DRG (Fig. S1).

**Figure 4:**
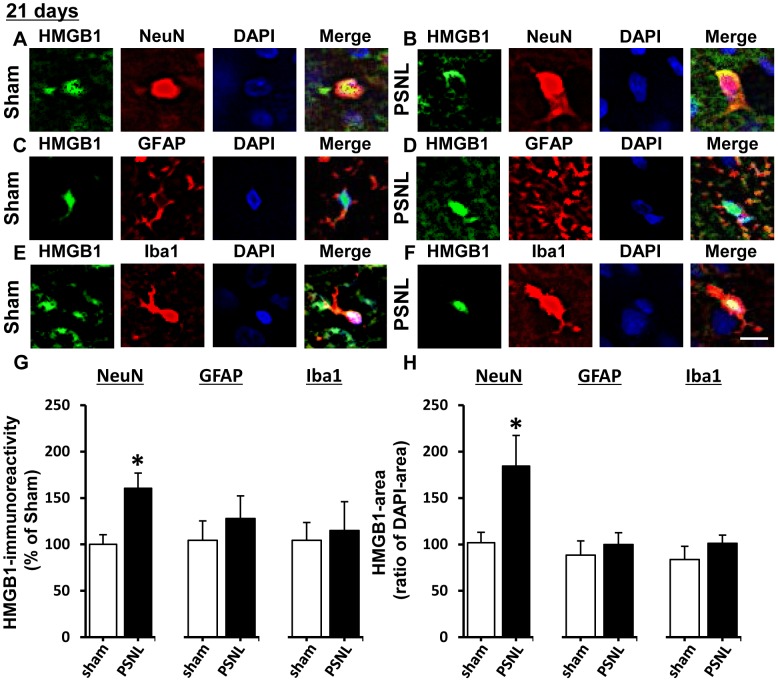
Distribution of HMGB1 expression in spinal dorsal horn neurons and glial cells. A-F. Double-labeling immunohistochemistry for HMGB-1 and NeuN (A, B, a marker for neurons), GFAP (C, D, a marker for astrocytes) or Iba1 (E, F, a marker for microglia) in the spinal dorsal horn of sham (A, C, E) and PSNL (B, D, F) rats 21 days following operation. DAPI was used to show nuclear localization. Scale bar  =  10 µm. G. Quantitative analysis of HMGB1 expression level in neurons, astrocytes and microglia at 21 days following operation. Data indicate the relative mean immunofluorescence intensity of a single cell. H. Quantitative analysis of the area of HMGB1 expression of neurons, astrocytes and microglia at 21 days following operation. Mean ratios of the area of HMGB1 immunofluorescence to the area of DAPI immunofluorescence are shown. Data are expressed as mean ± SEM. n = 6/group. * p<0.05 compared with sham group (Student’s t-test).

In the spinal dorsal horn, HMGB1 immunofluorescence intensity was enhanced at 21 days, but not 3 days, post-PSNL compared with dorsal horn from sham-operated rats (Fig. 3 B-E). Moreover, double-labeled immunostaining for HMGB1 and NeuN revealed that translocation of HMGB1 from the nucleus to the cytosol occurred in dorsal horn neurons on day 21 post-PSNL (Fig. 4A and B). In contrast, HMGB1 immunoreactivity in both astrocytes and microglia remained in the nucleus 21 days after PSNL (Figure 4C-F). In addition, immunoreactive intensity of the PSNL-induced increase in HMGB1 could be demonstrated only in the neurons (Figure 4G). Using this simple calculation, we have found a significant cytoplasmic translocation of HMGB1 (ratio greater than 100) in neurons but not in astrocytes and microglia (ratio about 100; Figure 4H).

### Effect of anti-HMGB1 monoclonal antibody on PSNL-evoked activation of glial cells and neurons

**Figure 5:**
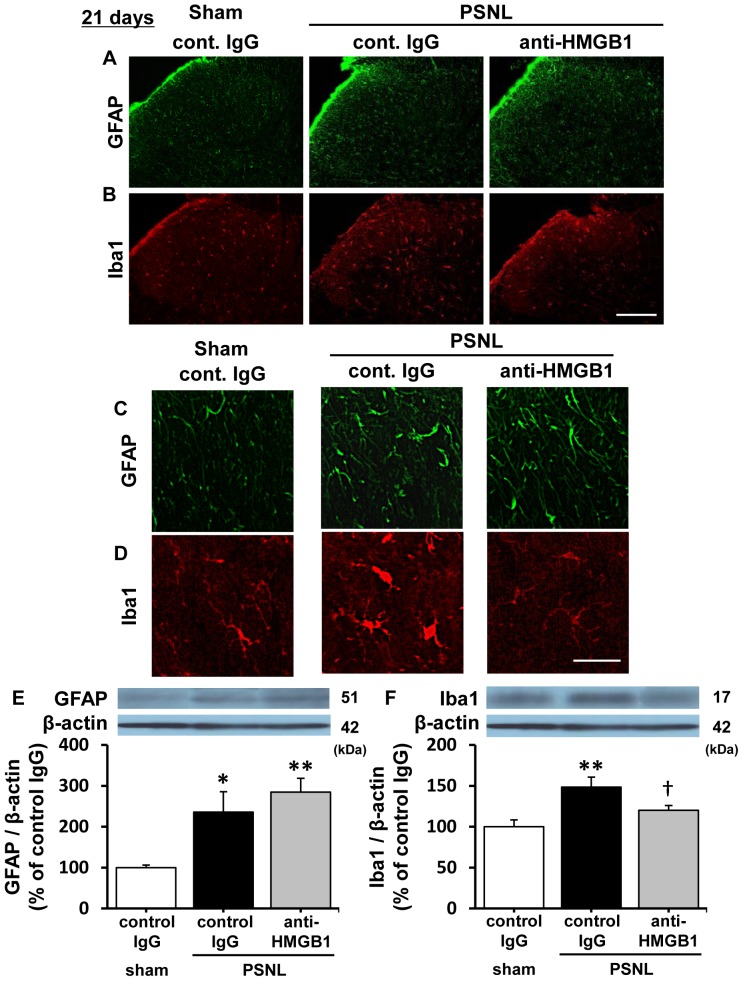
Effect of anti-HMGB1 monoclonal antibody on activation of spinal dorsal horn glial cells after PSNL. A, B. Immunofluorescence photomicrographs of spinal dorsal horn astrocytes (A) and microglia (B) from rats treated 2 hrs after with either anti-HMGB1 monoclonal antibody (2 mg/kg) or control IgG (2 mg/kg). Sham and PSNL rats were treated with antibody 21 days after operation. Scale bar  =  200 µm. C, D. High-power fields demonstrating morphological change of astrocytes (C) and microglia (D). Scale bar  =  20 µm. E, F. Levels of GFAP (E) and Iba1 (F) in the ipsilateral dorsal horn were quantified by Western blotting analysis. The panels indicate representative blots. The graph in lower panels indicates quantitative data for each blot. Protein levels were normalized to levels of β-actin. Data are expressed as mean ± SEM. n = 5/group. *, ** p<0.05, 0.01 compared with sham-control IgG group, † p<0.05 compared with PSNL-control IgG group (one-way ANOVA followed by Tukey-Kramer post hoc test).

The morphological changes in spinal microglia and astrocytes in the dorsal horn 21 days after PSNL, which could be considered the late maintenance phase of neuropathic pain in this model, were investigated by using immunohistochemistry. Twenty one days following PSNL, GFAP and Iba1-positive cells were increased in the ipsilateral spinal dorsal horn, whereas they were not increased in sham-operated rats (Fig. 5A and B). Intravenous injection with anti-HMGB1 monoclonal antibody (2 mg/kg) dramatically changed the morphologies of microglia, but not astrocytes, from ameboid state to ramified state (Fig. 5C and D). To quantify these levels of activation, protein levels of glial markers were examined by western blotting analysis after the treatment with anti-HMGB1 monoclonal antibody. The protein level of Iba1, but not GFAP, was significantly down-regulated 2 hr after treatment with anti-HMGB antibody (Fig. 5E and F). By contrast, injection with the control antibody had no effect on either Iba1 or GFAP expression in rats with a PSNL.

**Figure 6:**
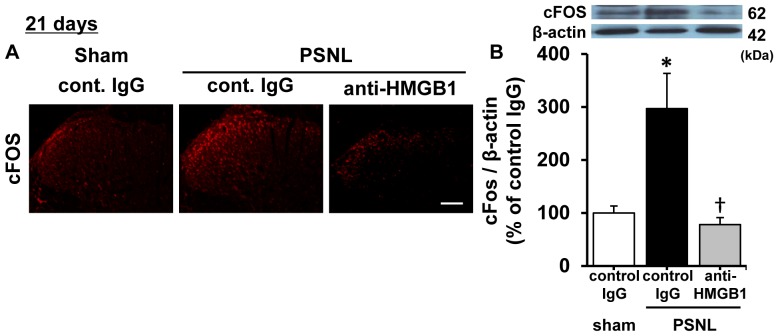
Effect of anti-HMGB1 monoclonal antibody on cFos expression in the spinal dorsal horn. A. Immunohistochemistry of spinal dorsal horn from rats 21 days following either a sham or PSNL surgery. The expression of cFos was evaluated 2 hrs after treatment with either anti-HMGB1 monoclonal antibody (2 mg/kg) or control IgG (2 mg/kg). Scale bar  =  200 µm. B. cFos in the ipsilateral dorsal horn was quantified by Western blotting. The panels above the graph indicate representative blots. The cFos data were quantified as a ratio of cFos to β-actin. Data are expressed as mean ± SEM. n = 5/group. * p<0.05 compared with sham-control IgG group, † p<0.05 compared with PSNL-control IgG group (one-way ANOVA followed by Tukey-Kramer post hoc test).

Next, to examine the degree of neural activity in the ipsilateral spinal dorsal horn, the protein level of cFos, an immediate early gene product and a marker of neuronal activation, was measured. A marked increase of cFos protein was obtained 21 days after PSNL. By contrast, no significant increase in cFos was observed following sham surgery. Furthermore, the increase of cFos in rats with a PSNL was significantly attenuated by treatment with an anti-HMGB1 monoclonal antibody (Fig. 6). No effect of the control antibody on cFos expression was observed in PSNL rats.

## Discussion

The current study demonstrated that a single systemic (i.v.) dose of anti-HMGB1 monoclonal antibody ameliorated PSNL-induced mechanical hypersensitivity for several hours during the maintenance phase (7, 14, 21 days), but not the induction phase (3 days), of neuropathic pain. The efficacy is likely due to neutralizing the effect of HMGB1 released from cells within the spinal dorsal horn. The current study demonstrated an increase of neuronal HMGB1 in the ipsilateral dorsal horn, which was translocated from the nucleus to the cytoplasm, indicating a potential source of extracellular HMGB1. Although HMGB1 was also expressed in glial cells, translocation of HMGB1 to the cytoplasm was not observed in glial cells, further suggesting that the source of secreted HMGB1 in spinal dorsal horn was from neurons. These results indicate that an increase of HMGB1 secretion from the spinal dorsal neurons is associated with the maintenance of the neuropathic pain state, and that a treatment against anti-HMGB1 that neutralizes HMGB1 could be a useful therapeutic strategy for ameliorating neuropathic pain.

It has been well established that HMGB1 is not only passively secreted during cellular necrosis by nearly all cell types that have a nucleus and serves to signal neighboring cells of ongoing damage but also actively secreted from neurons and immune cells such as macrophages and microglia following stimulation with lipopolysaccharide (LPS) or tumor necrosis factor (TNF)-α [3], [32]. Secretion of HMGB1 can trigger the release of numerous cytokines from inflammatory cells and lead to a positive-feedback autocrine loop, thereby leading to the further release of HMGB1 and maintenance of the inflammatory cascade [33], [34]. Previous studies have reported that pain-related behaviors in several preclinical models of chronic pain, including diabetic, cancer and neuropathic pain, were significantly attenuated by treatment with anti-HMGB1 antibody [14]–[16]. These studies suggest that the HMGB1 may be involved in the maintenance of chronic pain.

In the current study, a significant increase of HMGB1 in the spinal dorsal horn occurred 7 days following nerve injury and continued for an additional two weeks, even though significant hind paw mechanical hypersensitivity was observed beginning 3 days after nerve ligation. Moreover, the current findings indicate that HMGB1 originates from spinal dorsal horn neurons, and not glial cells, suggested by the translocation of HMGB1 to the cytoplasm from the nucleus in neurons. These data coincide with a previous study that demonstrated that HMGB1 was translocated and released from neurons, not glial cells, in the ischemic rat brain [9]. Thus, these data suggest that the HMGB1 secreted from dorsal horn neurons may play an important role in regulating “late” or maintenance phase of neuropathic pain. However, the neurochemical mechanism that mediates HMGB1 upregulation and secretion form spinal dorsal horn neurons in the late phase of neuropathic pain is unclear. One possible mechanism could be thought cytokines released from activated microglia and/or primary afferent neuron in the “early” or induction phase of neuropathic pain. Several studies have demonstrated that TNF-α is secreted from cultured microglia and DRG neurons, and that TNF-α stimulation modulates the expression and secretion of HMGB1 [27], [32], [35]. Furthermore, Wang et al. reported that the intrathecal injection of Etanercept, a recombinant TNF receptor (p75)-Fc fusion protein, inhibited the increased expression of HMGB1 by chronic constriction injury of the sciatic nerve [36]. In addition, HMGB1 release occurs considerably later than the secretion of typical pro-inflammatory mediators TNF-α and interleukin (IL)-1 [3], [34]. Thus, a possible sequence of events that leads to the maintenance of nerve injury-induced pain is that peripheral nerve injury evokes cytokine release from activated spinal microglia and/or primary afferent neurons, thereby leading to the upregulation and secretion of HMGB1 from spinal dorsal horn neurons.

The current study also showed that the nerve injury-induced mechanical allodynia, 21 days post-ligation, is significantly attenuated by i.v. anti-HMGB1 monoclonal antibody with a concurrent reduction of markers of neuronal and microglia activity. It is particularly interesting that acute i.v. administration of antibody effectively reduced mechanical allodynia. Intravenous injection would be a convenient means of delivery since currently available antibody drugs are injectables. Also, it is possible that the anti-HMGB1 monoclonal antibody reached the target tissue, the spinal cord, because of the significantly increased permeability of the BSCB following PSNL, thereby blocking HMGB1 function in both the spinal cord and in the circulation [30].

Pain hypersensitivity is thought to be due in part to altered processing of signals in the spinal nociceptive system [17], [18], [20], [21]. The current study confirms altered spinal neuronal activity following PSNL, by increased cFos immunoreactivity and this increase, along with cutaneous hypersensitivity, was diminished by anti-HMGB1 treatment. The present study also confirmed increased expression following a nerve injury of activation markers in microglia and astrocytes in spinal dorsal horn, but only Iba1 was suppressed by anti-HMGB1 treatment. Previous studies have reported that HMGB1-induced inflammation via the activation of inflammatory cells including macrophages and microglia [7], [8], [37], [38]. Moreover, Leclerc et al. reported that co-treatment with HMGB1/IL1β significantly increased the expression of microsomal prostaglandin E synthase-1 and cyclooxygenase-2 as well as prostaglandin E2 production compared to treatment with either HMGB1 or IL-1β alone [39]. Indeed, that anti-HMGB treatment did not completely reverse PNSL-induced hypersensitivity suggests that other substances released from spinal tissue following nerve injury may be crucial in the maintenance of the neuropathic pain state. Nonetheless, the data suggest that HMGB1, in concert with other substances, potentiates nociceptive processing following nerve injury at the level of the spinal cord via activation of microglia.

While the Iba1 and cFos were attenuated, the astrocytic marker GFAP was not attenuated, despite the fact that astrocytes are important for the maintenance of neuropathic pain [17], [18], [21]. Ren et al. reported that diabetes-induced astrocytic activation in the spinal dorsal horn is attenuated by intrathecal injection of anti-HMGB1 antibody [14]. Recently, studies have shown that astrocytes are activated by numerous substances, including cytokines/chemokines (e.g., TNF-α, IL-1β, IL-6 and IL-18), and neurotransmitters (e.g., glutamate, substance P, calcitonin gene-related peptide and ATP), in addition to HMGB1 [21]. Therefore, mediators in addition to HMGB1 could be crucial role in initiating and maintaining astrocyte activitation following nerve injury.

In conclusion, the current results indicate that nerve injury evokes HMGB1 release from dorsal horn neurons, and subsequent activation of spinal neuron and microglia by HMGB1 maintains the neuropathic pain state. Thus, blocking HMGB1 function, such as through an antibody, could prove to be a potent therapeutic strategy for the treatment of neuropathic pain.

## Supporting Information

Figure S1 **Expression level of HMGB1 mRNA in rat DRG after PSNL.** Expression levels of HMGB1 mRNA in the ipsilateral or contralateral DRG of sham (post-Sham 7-21 days) and PSNL (post-PSNL 7-21 days) rats at the indicated periods were measured by real-time PCR. Data are expressed as a ratio to sham values. Data are mean ± SEM. n = 4/treatment group.
(TIF)
