# Supplementary figures and images for: Neuropathic Pain in Rats with a Partial Sciatic Nerve Ligation Is Alleviated by Intravenous Injection of Monoclonal Antibody to High Mobility Group Box-1
Source: PLoS One. 2013 Aug 21;8(8):e73640. doi: 10.1371/journal.pone.0073640 (PMC3749159; doi:10.1371/journal.pone.0073640)

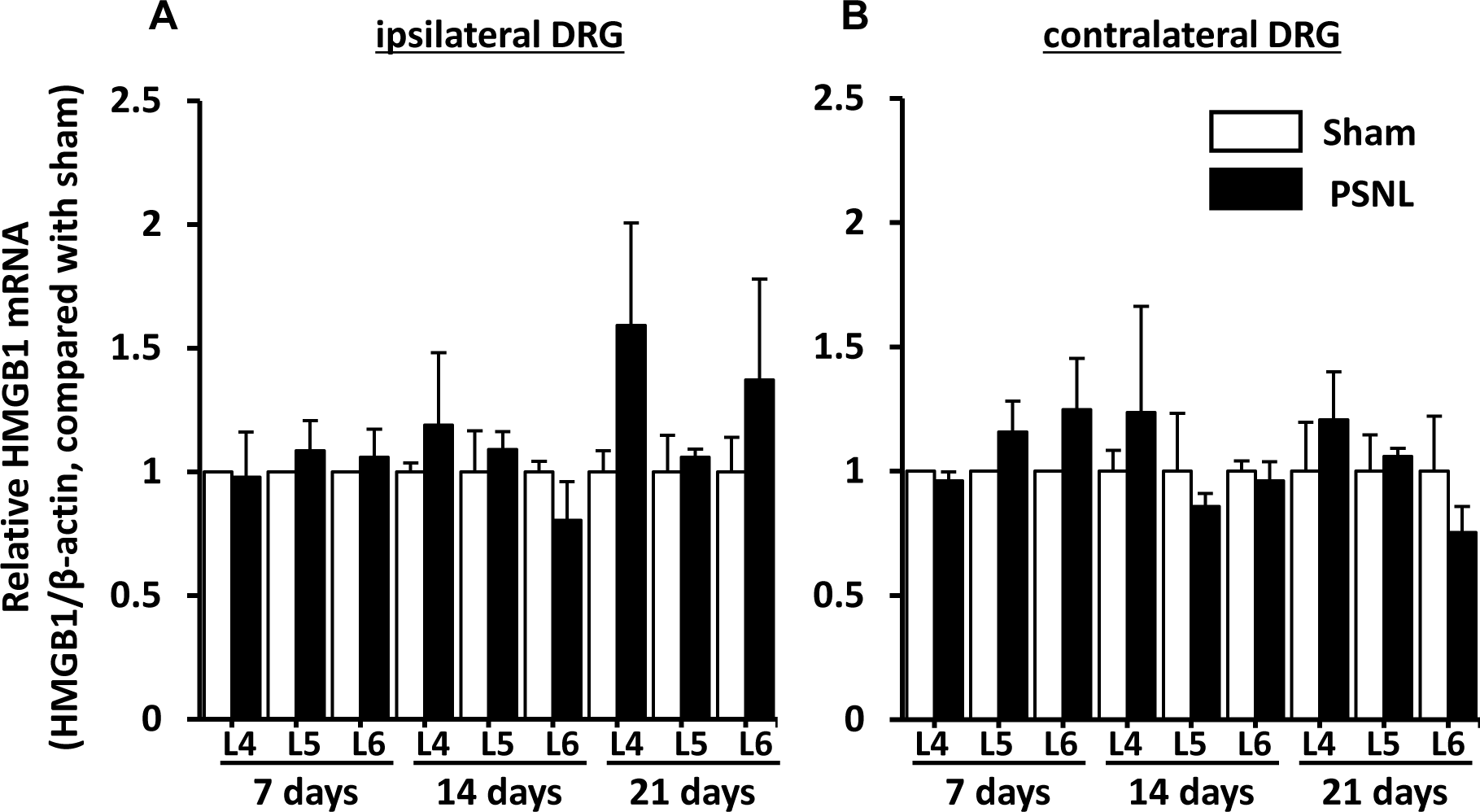

Supplement: Figure S1 — Expression level of HMGB1 mRNA in rat DRG after PSNL. Expression levels of HMGB1 mRNA in the ipsilateral or contralateral DRG of sham (post-Sham 7-21 days) and PSNL (post-PSNL 7-21 days) rats at the indicated periods were measured by real-time PCR. Data are expressed as a ratio to sham values. Data are mean ± SEM. n = 4/treatment group. (TIF) [file pone.0073640.s001.tif]
